# Comparison of Methods for the Histological Evaluation of Odontocete Spiral Ganglion Cells

**DOI:** 10.3390/ani10040683

**Published:** 2020-04-14

**Authors:** Tania Ramírez, Simona Sacchini, Yania Paz, Rubén S. Rosales, Nakita Câmara, Marisa Andrada, Manuel Arbelo, Antonio Fernández

**Affiliations:** 1 Veterinary Histology and Pathology, Institute of Animal Health and Food Safety (IUSA), Veterinary School, University of Las Palmas de Gran Canaria, Arucas, 35416 Las Palmas de Gran Canaria, Spain; simona.sacchini@ulpgc.es (S.S.); yania.paz102@alu.ulpgc.es (Y.P.); nakita.camara101@alu.ulpgc.es (N.C.); marisaana.andrada@ulpgc.es (M.A.); antonio.fernandez@ulpgc.es (A.F.); 2 Veterinary Epidemiology and Preventive Medicine, Institute of Animal Health and Food Safety (IUSA), Veterinary School, University of Las Palmas de Gran Canaria, Arucas, 35416 Las Palmas de Gran Canaria, Spain; ruben.rosales@ulpgc.es

**Keywords:** cetaceans, ear, decalcification, histology, spiral ganglion

## Abstract

**Simple Summary:**

Hearing is arguably the primary sensory and communication channel for cetaceans. The study of diverse physiological and pathological events involving this system, requires standardized and reliable protocols for processing valuable and scarce samples such as the ears of cetaceans. As part of our research, standardized tissue processing protocols were developed in order to improve our knowledge regarding the anatomy and pathological changes of this sensorial system, including the nervous structures of the ear. The results obtained suggest that ethylenediaminetetraacetic acid (EDTA)-based decalcifying solutions are key for correct evaluation of bony tissue structures such as the ear of cetaceans.

**Abstract:**

Cetaceans greatly depend on their hearing system to perform many vital activities. The spiral ganglion is an essential component of the auditory pathway and can even be associated with injuries caused by anthropogenic noise. However, its anatomical location, characterized by surrounding bony structures, makes the anatomical and anatomopathological study of the spiral ganglion a difficult task. In order to obtain high-quality tissue samples, a perfect balance between decalcification and the preservation of neural components must be achieved. In this study, different methodologies for spiral ganglion sample preparation and preservation were evaluated. Hydrochloric acid had the shortest decalcification time but damaged the tissue extensively. Both formic acid and EDTA decalcification solutions had a longer decalcification time but exhibited better preservation of the neurons. However, improved cell morphology and staining were observed on ears pretreated with EDTA solution. Therefore, we suggest that decalcifying methodologies based on EDTA solutions should be used to obtain the highest quality samples for studying cell morphology and antigenicity in cetacean spiral ganglion neurons.

## 1. Introduction

The hearing system is possibly the main sensory system of cetaceans, acting as their principal communication channel [1]. Cetaceans’ sense of hearing enables essential behaviors such as prey localization, predator detection, navigation, and the identification of conspecifics [2]. The rising levels of marine noise pollution appears to affect these behaviors, having been linked to stress, alterations in reproduction, gas and fat embolic syndrome, and hearing damage in cetaceans [3,4,5,6,7,8,9].

Well-preserved inner-ear samples from stranded cetaceans are scarce, so it is paramount to develop protocols for sample collection, preservation, and processing. In the present study, we will focus on the development of different strategies to accurately preserve the inner ear, more specifically, the spiral ganglion of cetaceans. The spiral ganglion is located in the cochlea and it is composed of type I and type II ganglion cells [10]. About 95% of the ganglion cells are classified as type I, which are connected to the inner hair cells in the organ of Corti. The remaining 5% correspond to type II ganglion cells, which connect to the outer hair cells of the organ of Corti [11]. These connections are produced through the peripheral processes of the ganglion cells. The central processes of these cells come together to form the acoustic portion of the cochlear nerve and end up the cochlear nucleus in the brainstem [12]. 

The anatomical location of the spiral ganglion is characterized by being surrounded by bony structures, which makes the anatomical and anatomopathological study of the spiral ganglion a difficult task [13]. Therefore, to carry out the morphological study of the spiral ganglion it is necessary to decalcify the bone that surrounds it. The decalcification consists of softening the tissue by removing inorganic calcium from the organic collagen matrix of bone tissue. This demineralization is carried out by chemical agents, either acids or chelating agents. While acids are used to form soluble calcium salts, chelating agents act by binding to calcium ions [14,15]. 

In order to find the most suitable decalcifying agent, four different decalcifying solutions were evaluated. Ears from cetaceans stranded in the Canary Islands were used for the evaluation of the decalcification procedure by measuring the decalcification time, ease of sectioning, and morphological conservation of the tissue. In addition, antigenic preservation was evaluated by immunohistochemistry.

## 2. Materials and Methods 

### 2.1. Tissue Specimens

Six ears from five cetaceans, representing three species (*Delphinus delphis* (*n* = 2), *Kogia breviceps* (*n* = 1), and *Tursiops truncatus* (*n* = 2)), were studied. All samples were obtained from dead stranded cetaceans in the Canary Islands. Environmental variables such us weather conditions, tide, cetacean species, and size complicate the task of determining the exact time of death of the animals sampled. For that reason, animals were assigned a conservation code at the time of necropsy [16] based on both the external and internal conditions of the animal. The elapsed time between the localization of the stranded animal and the necropsy was variable (Table 1). In addition, due to logistics issues, one of the animals was previously frozen and another one was refrigerated prior to necropsy.

### 2.2. Tissue Fixation

Once extracted, the ears were perfused through the oval and round window with a fixative solution (4% neutral-buffered formalin (NBF)). Subsequently, the samples remained immersed in 4% NBF for 2–22 days at room temperature [17]. 

### 2.3. Decalcification

Once the samples were fixed in NBF, they were immersed in a decalcifying solution. Four decalcifiers were evaluated and included Histofix^®^ decalcifier 3 (Panreac Química S.L.U., Barcelona, Spain), containing 10% hydrochloric acid, 15% formic acid (18.6 g of formic acid in 100 mL of 4% formaldehyde solution), 10% EDTA (10 g of EDTA per 100 mL of phosphate-buffered saline (PBS), adjusted to pH 7.2–7.3 with NaOH), and 20% EDTA (20 g of EDTA per 100 mL of PBS, adjusted to pH 7.2–7.3 with NaOH).

Ears were placed into the assigned decalcifying solutions (Table 1) and kept at room temperature (RT). The decalcifying solutions (except hydrochloric acid) were changed on a weekly basis and the total decalcification time was recorded. 

Decalcification time, preservation of morphological features (evaluated by histochemistry), and antigenicity (evaluated by immunohistochemistry) were studied. 

### 2.4. Tissue Processing

After decalcification, samples were routinely processed, embedded in paraffin, and sectioned to a thickness of 3–5 µm. Briefly, tissue samples were placed in 10% NBF for 3 h. After that, tissue samples were dehydrated through a series of graded ethanol solution as follows: 70% ethanol for 1 h; two incubation steps in 96% ethanol for 1 h each; 100% ethanol for 1 h; two incubation steps in 100% ethanol for 1.5 h each; xylene for 1 h; and two incubation steps in xylene for 1.5 h each. All these steps were performed at room temperature. Finally, samples were placed in paraffin wax at 59 °C for two incubation steps of 1.5 h each. In addition, samples from the two *Tursiops truncatus* (*n* = 2) were used for the preparation of cryosections. Samples were washed in PBS and immersed in a 30% sucrose at 4 °C, until the samples reached the bottom of the container. After that, samples were removed from the sucrose solution and embedded in OCT^®^ (Optimal Cutting Temperature). Then, 15-µm cryosections were obtained in a cryostat at −25 °C, collected on glass slides and air-dried. 

### 2.5. Histochemical Staining

All sections were stained with HE solution. To carry out the staining, paraffin was removed by immersing the tissue through two changes of xylene, for 2 min each. Sections were then re-hydrated with 100% ethanol twice for 2 min each, followed by 70% ethanol for 2 min and with deionized water three times for 3 min each. After deparaffinization and rehydration, samples were stained using Harris hematoxylin solution for 14 min, followed by a 1-min rinse in deionized water. Then, samples were differentiated in hydrochloric ethanol through four brief dips followed by a 1-min wash in deionized water. Bluing was performed in ammoniacal water through 15 dips. Specimens were washed in running tap water for 15 min and counterstained in eosin solution for 4 min. Finally, sections were dehydrated with 96% ethanol (twice) and 100% ethanol (twice), for 2 min each, and cleared up with xylene three times for 3 min each. 

Thionine and cresyl violet were used for the staining of the Nissl substance (regions with granular endoplasmic reticulum, ribosomes and polysomes, where intense protein synthesis occurs [18]) present in neurons. 

Thionine staining was performed by immersing the paraffin sections in a solution composed of 50% ethanol and chloroform in equal parts. After that, samples were re-hydrated through a series of graded ethanol baths: 100% ethanol, 96% ethanol, 90% ethanol, 80% ethanol, 70% ethanol, 50% ethanol, and deionized water for 3 min each. Then the samples remained submerged in 0.125% thionine for 10–15 min. Dehydration was carried out in 5% ethanol, 70% ethanol, 80% ethanol, 90% ethanol, 96% ethanol (10 brief dips each), and 100% ethanol and xylene for 10 min each.

For cresyl violet staining, sections were first immersed in 100% ethanol twice for 2 min each and in xylene twice for 2 min each before being stained in cresyl violet stain solution for 20 min. Subsequently, samples were subjected to four dips of 95% ethanol and two immersions in 100% ethanol for 2 min each, as well as of xylene twice for 2 min each.

Cryosections were processed as described above with some protocol modifications. HE staining consisted of a four step protocol. Samples remained 1 min in the Harris hematoxylin solution. Then, they were briefly washed with deionized water and then immersed for 30 s in eosin solution, followed by a final wash with deionized water. Cresyl violet staining was performed using an initial 2-min incubation step in 100% ethanol at room temperature, followed by cresyl violet staining solution, ethanol (95%–100%) and xylene incubation steps as described above. Cryosections stained with thionine were processed as described above.

Both paraffin-embedded samples and the cryosections were mounted after each staining procedure using DPX mounting medium (Panreac). HE-stained cryosections were mounted in aqueous medium (Dako Faramount Aqueous Mounting Medium). 

### 2.6. Immunohistochemical Staining

Three primary antibodies were used to evaluate tissue antigenic preservation. Anti-hsp70 antibody (Abcam, ab6535, Abcam, Cambridge, UK) is believed to maintain the native folding of proteins under stress conditions [19] and has been linked to cases of ischemia [20]. The other two antibodies, anti-calretinin (Swant, 6B3, Swant Inc, Marly, Switzerland) and anti-nitric oxide synthase (nNos) antibody (Chemicon, ab5380, Merck-Millipore, Darmstadt, Germany), can be observed in physiological conditions. Calretinin activity has been associated with potential neuroprotective activity [21,22] and protection against noise trauma [23]. nNos activity has been linked to the control of cerebral blood flow [24], as well as a potential role in ischemia [25].

Before immunostaining, all paraffin sections were dewaxed in xylene and rehydrated through graded series of alcohol to water. Endogenous peroxidase was blocked after incubation with 3% H_2_O_2_ in methanol for 30 min. Heat pretreatment was required for the monoclonal antibody hsp70. In this case, citrate buffer pH 6 was used at 90–95 °C for 10 min. Prior to the specific antibody addition, non-specific binding sites were blocked using goat serum for those tissue samples incubated with the polyclonal antibody, and horse serum for those samples incubated with monoclonal antibodies. Incubation with goat and horse serum was performed at 1:10 dilution for 90 min at room temperature (RT). Tissue sections were incubated with primary antibody (nNos at 1:300 dilution, calretinin and hsp70 at 1:100 dilution) at 4 °C overnight. After that, sections were kept at RT for 1 h and then washed several times with phosphate-buffered saline and incubated with the corresponding secondary antibodies (Biotinylated anti-rabbit IgG in the case of nNos and biotinylated anti-mouse IgG for calretinin and hsp70) at 1:200 dilution for 1 h at RT. Specimens were incubated with 4% Avidin-Biotin Complex (VECTASTAIN^®^ Elite^®^ ABC-HRP Kit, Vector Laboratories, Burlingame, CA, USA) for 1 h in the dark followed by the addition of 3-amino-9-ethylcarbazole (AEC; Vector, SK 4200, Vector Laboratories, Burlingame, CA, USA) chromogen and counterstaining with Mayer´s hematoxylin. At the end of all the staining steps, the sections were washed with deionized water and mounted in aqueous medium (Dako Faramount Aqueous Mounting Medium, Dako, Glostrup, Denmark).

Cryosections were washed with PBS and incubated for 30 min at RT with 1% H_2_O_2_ in PBS for blocking endogenous peroxidase. After several washes with PBS, samples were incubated for 2 h at RT with PBS containing 10% goat or horse serum to prevent non-specific labeling. Incubation with primary antibody (nNos at 1:300 dilution, calretinin and hsp70 at 1:100 dilution) was performed for 48 h at 4 °C. Cryosections remained for 1 h at RT and were then washed three times with PBS. Tissue sections were exposed to secondary antibodies at 1:200 diluted for 2 h. Samples were then processed as described above using 4% Avidin-Biotin Complex (VECTASTAIN^®^ Elite^®^ ABC-HRP Kit) for 1 h in the dark followed by the addition of 3-amino-9-ethylcarbazole (AEC; Vector, SK 4200) chromogen and counterstaining with Mayer’s hematoxylin.

## 3. Results

The time of decalcification, preservation of macro and microscopic morphology, and antigenicity conservation for each decalcifying agent can be seen in Table 2.

### 3.1. Decalcification Time

Decalcification time varied according to the reagent used. Two days were required for full decalcification in Histofix^®^ decalcifier 3, seven days in 15% formic acid, 28 days in 10% EDTA, and 25–31 days in 20% EDTA.

### 3.2. Preservation of Macroscopic Morphology

The macroscopic morphology of samples processed with EDTA or Histofix^®^ decalcifier 3 was highly preserved after the decalcification process. Conversely, the morphology of the specimen decalcified using 15% formic acid was extensively altered in the process. Therefore, this sample partially lost its macroscopic morphology, preventing cresyl violet and immunohistochemical staining of all the 15% formic acid-decalcified specimen.

### 3.3. Preservation of Microscopic Morphology

Tissue microscopic morphology preservation was evaluated by hematoxylin and eosin, thionine, and cresyl violet staining.

All sections showed a good stain uniformity after hematoxylin and eosin staining for the general evaluation of tissue morphology. Nevertheless, EDTA-decalcified cryostat-processed tissue samples exhibited the greatest tissue morphology and stain uniformity.

**Figure 1:**
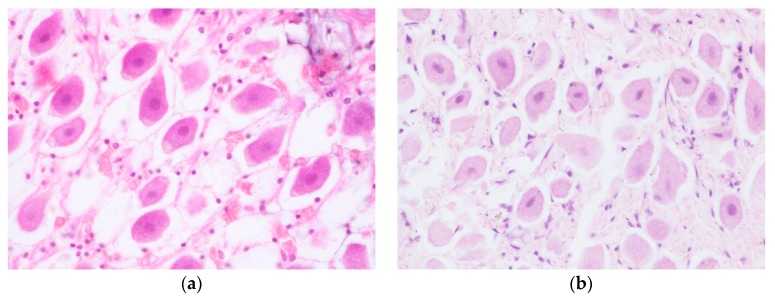

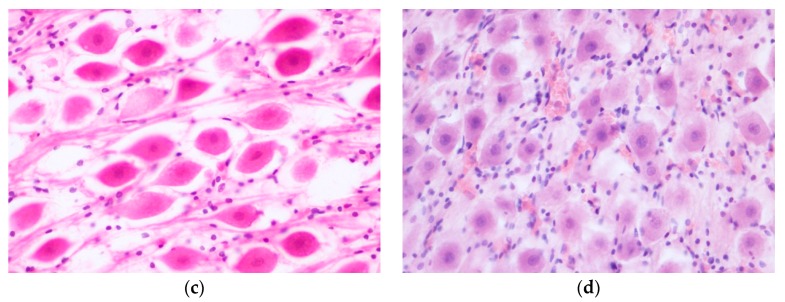
Spiral ganglion, hematoxylin and eosin staining. Panels contain the samples obtained from the different descaling methods: (**a**) Histofix decalcifier 3^®^ (magnification 60×); (**b**) 15% formic acid (magnification 60×); (**c**) 10% EDTA (magnification 60×); and (**d**) 20% EDTA (magnification 40×).

The neuronal architecture was preserved with all the decalcifying protocols evaluated. However, some variation in the neuronal staining performance was observed, mainly affecting basophile structures (nucleus, nucleolus, and satellite glial cells). Ears decalcified with Histofix^®^ decalcifier 3 (Figure 1a) and 10% EDTA (Figure 1c) showed high eosin staining and weak basophilia, as well as faded nuclear and nucleolar staining. Basophilia appeared better preserved in the tissue decalcified with 15% formic acid (Figure 1b) or 20% EDTA (Figure 1d). In both cases, the nuclei could be clearly visualized. Regarding the nucleoli, 15% formic acid processed sample presented a slightly weaker staining, while 20% EDTA displayed the best staining performance for this structure.

**Figure 2:**
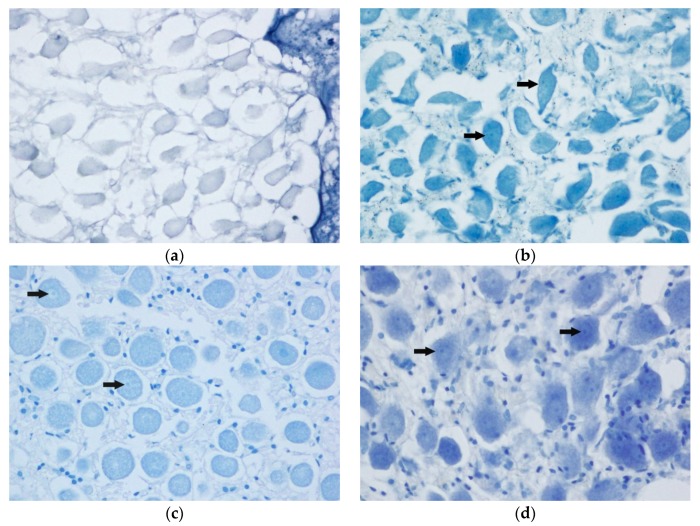
Spiral ganglion, thionine staining. Following images correspond to a specific staining of the Nissl substance (arrows) for each descaling method: (**a**) Histofix decalcifier 3^®^ (magnification 60×); (**b**) 15% formic acid (magnification 60×); (**c**) 10% EDTA (magnification 60×); and (**d**) 20% EDTA (magnification 60×).

**Figure 3:**
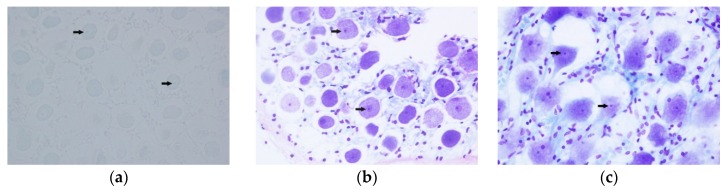
Spiral ganglion, cresyl violet staining. Images show the Nissl substance (arrows) present in the spiral ganglion neurons of the ears processed with the different decalcification protocols: (**a**) Histofix decalcifier 3^®^ (magnification 60×); (**b**) 10% EDTA (magnification 60×); and (**c**) 20% EDTA (magnification 60×).

Thionine and cresyl violet staining presented similar results, displaying a poor efficacy for Histofix^®^ decalcifier 3-decalcified samples, with no complete staining of the tissue in those specimens. Using this decalcifier, thionine stained samples did not provide evidence of cell structure (Figure 2a), while those stained with cresyl violet displayed a subtle definition of the neuronal structure and a very weak labelling of nucleoli and glial satellite cells (Figure 3a). 

The 20% EDTA-decalcified samples exhibited the highest degree of conservation of the Nissl substance (Figure 2d and Figure 3c), which is considered as good evidence of tissue preservation (neuronal structure, nucleoli and glia satellites cells). The 15% formic acid-decalcified sample also displayed a good staining efficacy using thionine, with a very good conservation of the neuronal architecture and clearly visible nucleoli (Figure 2b). However, the satellite glial cells were poorly differentiated in the tissue sample processed using this decalcifier. Cresyl violet could not be used for staining this sample due to tissue alteration, as described above. Sample treated with 10% EDTA displayed a fair staining of the Nissl substances using thionine (Figure 2c), while cresyl violet presented the best staining efficacy for this decalcifier (Figure 3b).

### 3.4. Conservation of Antigenicity

**Figure 4:**
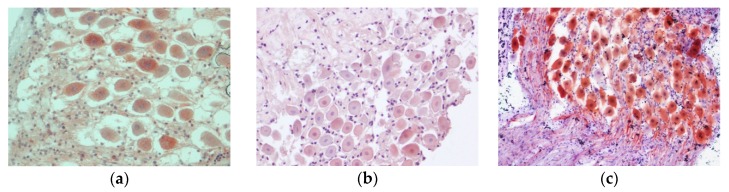
Spiral ganglion, immunohistochemical staining. The histological images show the results of the different immunohistochemical techniques with their corresponding antibodies and descaling agent: (**a**) Histofix decalcifier 3^®^ (anti-hsp70 antibody; magnification 40×); (**b**) 10% EDTA (anti-nitric oxide synthase antibody; magnification 40×); and (**c**) 20% EDTA (anti-calretinin; magnification 20×).

All samples subjected to immunohistochemical techniques presented a good antigenicity preservation. The 20% EDTA-decalcified cryostat-processed samples showed the greatest degree of antigen preservation, where the labelling of the antibody could be found in both the neuronal bodies and the axons, which will later form the cochlear nerve (Figure 4c). The immunolabeling of the neuronal bodies was weaker for samples processed using Histofix^®^ decalcifier 3 (Figure 4a) and 10% EDTA (Figure 4b), with a slightly better distribution of immunolabeling in neuronal somas for samples treated with the former. The sample processed with 15% formic acid could not be processed for immunohistochemical staining due to tissue alteration. 

## 4. Discussion

As a result of the anatomical nature of the spiral ganglion, that is, it being covered by a bone component, the development of optimal decalcifying strategies is paramount for the appropriate histopathological study of this tissue. As it is difficult to obtain ears from stranded cetaceans in a good state of preservation, it is important to develop protocols for optimal sample preservation and processing. Several studies have been developed comparing decalcifying agents to date. We used four protocols with four different decalcifiers: hydrochloric acid (Histofix^®^ decalcifier 3), 15% formic acid, and 10% and 20% EDTA. After decalcifying the samples, in terms of the decalcifying agent, we next valued their ability to decalcify samples in the shortest time, the morphological preservation by histochemistry staining, and the antigenicity preservation by immunochemistry staining. In general, acceptable results were obtained from all solutions, but 20% EDTA offered better results. 

With respect to the time it took the samples to decalcify, the Histofix^®^ decalcifier 3 was the fastest and the slowest were 10% and 20% EDTA. The samples processed with 20% EDTA came from two calves of *Tursiops truncatus*, so it is possible that if they had been adults, the ears would have been slower to decalcify [26]. Results from the present study concur with other studies in which EDTA took more time than hydrochloric acid. However, the time it took the samples to decalcify in formic acid was similar to EDTA, which is inconsistent with other studies carried out with the same decalcifying agent [27,28,29].

The samples that better maintained their macroscopic morphology, and thus allowed good handling when processing, were those treated with EDTA and hydrochloric acid. On the other hand, the decalcified ears with 15% formic acid maintained a poor macroscopic morphology and were friable when handling them. The results obtained concur, completely or partially, with several studies carried out in which the authors value the ease of handling and conservation of the morphology of decalcified osteological material with different agents [27,28,30,31]. Choube et al. observed that the samples that were processed with formic acid and had heat applied were sectioned better [32]. In another study, they determined that the samples processed with EDTA were those that presented a worse structure when sectioning the samples after decalcification [27].

Although ears processed with formic acid, hydrochloric acid, and 10% EDTA preserved good cell morphology, evaluated by hematoxylin and eosin staining, the result with 20% EDTA was better. Similar results were obtained in previous studies [27,28]; however, in one of them, the formic acid showed some deficiencies in this aspect [31]. Nonetheless, some authors have described potential over-decalcification artefacts in EDTA-decalcified cetaceans ears [33]. We also used the Nissl substance staining to evaluate the conservation of the cellular structure. In this case, the best results were obtained with 20% EDTA and the worst were obtained with Histofix^®^ decalcifier 3. Kristensen described that the most difficult part of his study had been getting a good stain of the Nissl substance in the spiral ganglion [34].

The conservation of antigenicity in our research was better preserved with 20% EDTA compared with hydrochloric acid and 10% EDTA. In most studies, EDTA is the decalcifying agent of choice to perform immunohistochemical techniques [28,35,36]. The decalcified samples with 20% EDTA that were later processed with the cryostat offered the best immunohistochemical results; this concurs with the results obtained by other authors [37]. This could be because samples embedded in paraffin must be exposed to heat and xylene to remove paraffin, therefore losing some of the antigenicity of the tissue [38]. However, limitations in the number of available samples did not allow us to fully compare paraffin-embedded and cryostat-derived samples using the protocols described, and further studies should be performed in order to examine these different tissue evaluation strategies. Despite what has been previously commented on EDTA and its ability to preserve the antigenicity of the tissues, in our study, decalcified samples with 10% EDTA did not offer very good results; this was most likely due to the conservation status of the animal when the necropsy was performed.

As the soft tissue of the ear deteriorates only a few hours after death [33], the degree of decomposition of the animal and a rapid tissue fixation are the most relevant factors leading to a good analytical outcome of the sample. Tissues processed with Histofix^®^ decalcifier 3, 15% formic acid, and 20% EDTA came from animals that were classified at the time of necropsy with a degree of preservation ranging from fresh to very fresh. However, the ear treated with 10% EDTA was sourced from an animal with moderate signs of decomposition. Although a higher degree of decomposition was observed in this sample, both thionine and cresyl violet staining and immunolabeling showed acceptable results.

## 5. Conclusions

Obtaining samples from cetaceans in a good state of conservation is very complicated, so having standardized decalcification protocols is critical to allow the correct evaluation of tissue samples. Due to this, the sample decalcifying time should not be considered as a limiting factor as it will ensure an optimal tissue quality for further evaluation. Therefore, although EDTA-based solutions are the slowest decalcifying agents, they offer the best results and should be considered the decalcifying solutions of choice; specifically, samples decalcified with EDTA at 20% and processed in cryostat in offer the best balance between tissue and antigenicity conservation. Despite the difficulty in handling of tissue processed with 10% formic acid, promising results were obtained when using this decalcifier. However, we suggest that future studies should be aimed at evaluating lower concentration formic acid solutions. The positive labeling of the spiral ganglion obtained using the tissue processing protocols described as part of our research represents an optimal starting point for future research efforts focused on the physiological and pathological status of stranded cetaceans. Although useful results have been obtained as part of our research, it is important to continue optimizing tissue processing protocols in order to obtain as much information from tissue samples of cetaceans as possible. These samples, usually scarce and altered by conservation issues, are of central importance for understanding the pathological processes affecting these wild animals.

## Figures and Tables

**Table 1:** Information about animals and descaling protocols included in the study.

Animal	Ears (n)	Age	Type of Decalcifying	Conservation Code	Time Elapsed Between Stranding and Necropsy
*Kogia breviceps* (1/1)	2	Adult	10% hydrochloric acid (Histofix^®^ Decalcifier 3)	Fresh	<24 h
*Delphinus delphis* (1/2)	1	Juvenile	15% formic acid	Fresh	38 days (frozen animal)
*Delphinus delphis* (2/2)	1	Juvenile	10% EDTA	Moderate decomposition	2 days (chilled animal)
*Tursiops truncatus* (1/2)	1	Calf	20% EDTA	Extremely fresh	<24 h
*Tursiops truncatus* (2/2)	1	Calf	20% EDTA	Extremely fresh	<24 h

**Table 2:** Performance characteristics of the different decalcifying agents used in the study. NT: not tested. HE: hematoxylin and eosin staining.

Decalcifying Agents	Decalcification Time (In Days)	Macroscopic Morphology Preservation	Microscopic Morphology Preservation		Antigenicity Conservation
			HE	Thionine/Cresyl Violet	
Histofix^®^ Decalcifier 3	2	Very good	Good	Poor/poor	Good
15% Formic Acid	7	Poor	Good	Good/NT	NT
10% EDTA	28	Good	Poor	Fair/food	Fair
20% EDTA	25–31	Good	Very good	Very good/very good	Very good
